# Sizes and Shapes of Perivascular Spaces Surrounding Murine Pial Arteries

**DOI:** 10.21203/rs.3.rs-2587250/v1

**Published:** 2023-02-17

**Authors:** Nikola Raicevic, Jarod M. Forer, Antonio Ladrón-de-Guevara, Ting Du, Maiken Nedergaard, Douglas H. Kelley, Kimberly Boster

**Affiliations:** 1 Department of Mechanical Engineering, University of Rochester, Rochester, USA.; 2 Center for Translational Neuromedicine and Department of Neuroscience, University of Rochester Medical Center, Rochester, USA.; 3 Department of Biomedical Engineering, University of Rochester, Rochester, USA.; 4 School of Pharmacy, China Medical University, China.

**Keywords:** Perivascular space, Periarterial space, Cerebrospinal fluid, Hydraulic resistance

## Abstract

**Background:**

Flow of cerebrospinal fluid (CSF) through brain perivascular spaces (PVSs) is essential for the clearance of interstitial metabolic waste products whose accumulation and aggregation is a key mechanism of pathogenesis in many diseases. The PVS geometry has important implications for CSF flow as it affects CSF and solute transport rates. Thus, the size and shape of the perivascular spaces are essential parameters for models of CSF transport in the brain and require accurate quantification.

**Methods:**

We segmented two-photon images of pial (surface) PVSs and the adjacent arteries and characterized their sizes and shapes of thousands of cross sections from 14 PVS segments in 9 mice. Based on the analysis, we propose an idealized model that approximates the cross-sectional size and shape of pial PVSs, closely matching their area ratios and hydraulic resistances.

**Results:**

PVS size only approximately scales with vessel size, and the ratio of PVS-to-vessel area varies widely across the thousands of cross sections analyzed. The hydraulic resistance per unit length of the PVS scales with the PVS cross-sectional area, and we found a power-law fit that predicts resistance as a function of the area. Three idealized geometric models were compared to PVSs imaged in vivo, and their accuracy in reproducing hydraulic resistances and PVS-to-vessel area ratios were evaluated. The area ratio was obtained across thousands of different cross sections, and we found that the distribution peaks for the original PVS and its closest idealized fit (polynomial fit) were 1.12 and 1.21, respectively. The peak of the hydraulic resistance distribution is 1.73×10^15^ Pa s/m^5^ and 1.44×10^15^ Pa s/m^5^ for the segmentation and its closest idealized fit, respectively.

**Conclusions:**

PVS hydraulic resistance can be reasonably predicted as a function of the PVS area. The proposed polynomial-based fit most closely captures the shape of the PVS with respect to area ratio and hydraulic resistance. Idealized PVS shapes are convenient for modeling, which can be used to better understand how anatomical variations affect clearance and drug delivery transport.

## Background

**Figure 1:**
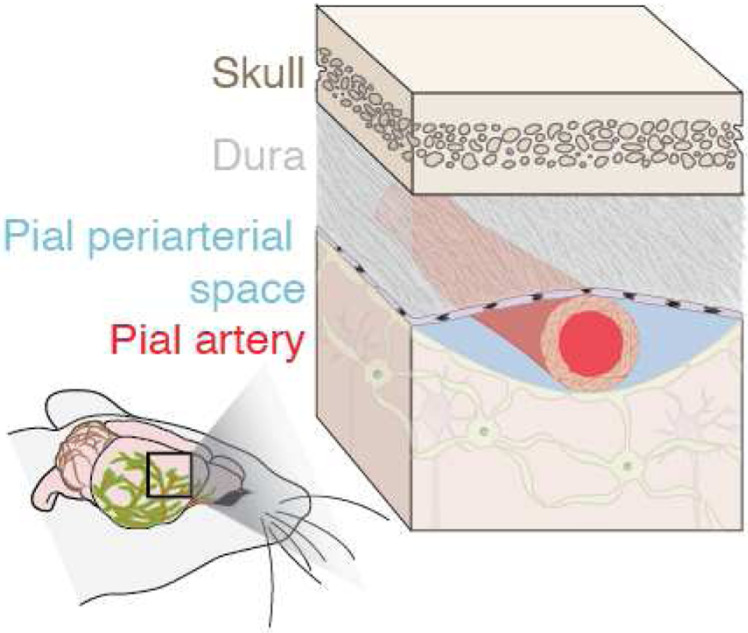
We characterize the size and shape of perivascular spaces (PVSs) adjacent to murine pial arteries, which serve as channels for flowing cerebrospinal fluid.

Flow of cerebrospinal fluid (CSF) through perivascular spaces (PVSs) surrounding pial (surface) vessels (Figure 1) in the brain is part of the brain’s transport system responsible for clearing metabolic waste including intraparenchymal extracellular macromolecules [1–3]. The perivascular spaces are pia-lined, fluid-filled structures surrounding the walls of arteries, arterioles, veins, and venules, as depicted in Figure 1 [4]. These spaces are bounded by the arachnoid mater, pia mater, and possibly by the recently-observed subarachnoid lymphatic-like membrane, where the intracranial vessels, or pial vessels, are located on the surface of the brain [5, 6]. The brain’s CSF transport system is responsible for waste clearance and distribution of compounds including glucose, lipids, amino acids, growth factors, and neuromodulators from brain parenchyma [3, 7–14]. The failure or abnormal functioning of the brain’s transport system has been linked to hypertension, atherosclerosis, stroke, aging, and neurodegenerative diseases like Alzheimer’s [1, 8, 15–17].

The size and shape of pial PVSs directly affect the flow of CSF into the extracellular spaces and transport of solutes (e.g. waste and nutrients) in the brain, making accurate knowledge of the morphology of these spaces important for modeling [18]. Effective modeling requires PVS shapes to be known precisely because parameters like hydraulic resistance are very sensitive to channel size. Several previous studies modeled the PVS as concentric circular annuli [19–21], but Tithof et al. [18] showed that the shape of the PVS is often eccentric, having lower hydraulic resistance (i.e., resulting in a faster flow of CSF for a given driving pressure). They proposed modeling the pial PVS as an elliptical annulus to account for the observed eccentricity. PVS size and shape are also important because their abnormalities are associated with stroke, hypertension, and white matter hyperintensities, which result from ischemia and cause cognitive decline [22]. Enlarged perivascular spaces are also associated with reduced waste clearance and, thus, may serve as an imaging marker for disease initiation and progression ([23–26]).

Previous works have quantified various aspects of PVS size. Bedussi et al. [27] showed that the size of the PVS scales approximately linearly with the size of its vessel and that the morphology of human and rodent PVSs is qualitatively similar. However, they did not report an average PVS-to-vessel area ratio or describe the shape. Schain et al. [2] characterized the PVSs around arteries and veins in mice and reported that the average cross-sectional area of the PVS was 305 ± 140 *μ*m^2^ near arteries and 90.9 ± 28 *μ*m^2^ near veins. The average PVS-to-vessel area was 1.26 for arteries compared to 0.13 for veins. They also found that PVSs varied in size depending on location, with larger PVSs in locations with multiple blood vessels or blood vessel bifurcations. However, they did not characterize this variation and only reported a single average area measurement for arterial PVSs. Mestre et al. [28] reported the cross-sectional PVS-to-vessel area ratio for pial vessels to be 1.4 for N = 13 mice, but they found considerable variation between samples. They also showed that PVS area changes drastically with death and fixation, explaining why measurements of in vivo PVS areas were much larger than previously supposed. These studies contributed to our understanding of PVS size, but none included more than tens of cross sections in their analysis, and beyond characterizing the standard deviation, they did not report on variation in PVS size, though it is clearly large, based on the report of Schain et al. [2].

Tithof et al. [18] characterized PVS shapes in five locations: three pial and two penetrating PVSs. They idealized the shapes of the vessels and PVSs as circles and ellipses, respectively, and reported how the hydraulic resistance of the idealized geometries varied with shape, demonstrating that the in vivo PVS shapes were similar to the shape with minimum resistance for a given area ratio. They also showed that approximating a PVS as a concentric annulus overestimates the hydraulic resistance. They showed that for an elliptical annulus, the minimum hydraulic resistance for a given periarterial cross-sectional area often occurs when the elliptical outer boundary intersects the circular inner boundary, creating two lobes on either side of the artery, and that, for the cross sections they examined, in vivo shapes were close to those which minimized the hydraulic resistance for a given area ratio. They also showed that, if both inner and outer boundaries are nearly circular, as often occurs around penetrating arterioles, the hydraulic resistance is minimum when eccentricity is large.

Vinje et al. [5] obtained a single cross-sectional image of a pial arterial PVS and a pial venous PVS from optical coherence tomography. They idealized the shape of the PVS as a long narrow section with the bulk of the area concentrated around the vessel. With simulations, they showed that the size and shape of the PVS can significantly change the mass and momentum transport: velocity and tracer distributions di ered from arterial to venous PVSs and from idealized to realistic geometries.

Both Tithof et al. and Vinje et al. [5, 18] clearly showed that the shape of the PVS plays an important role in transport. However, both works were based on only a few cross sections, and neither provided strong justification for its choice of idealized PVS shape, or made an attempt to rigorously quantify PVS shape.

An idealized model of PVS sizes and shapes, built by characterizing a statistically significant number of in vivo images, could improve the accuracy of future attempts to predict flows and solute transport in PVSs. Such a model should be complex enough to capture salient features of PVS geometry but simple enough to be easily comprehensible and computationally inexpensive. Its simplifications should be explained, and their impact on key PVS parameters like hydraulic resistance should be quantified. To be broadly applicable, an idealized model should be based on a known and statistically meaningful set of measurements, though only a few in vivo PVS measurements have been published so far.

In this paper, we characterize the size and shape of thousands of different pial perivascular space cross sections based on 3D two-photon images from 14 locations (different PVS segments) in 9 different wild-type mice. After segmenting the images to determine in vivo PVS shapes, we approximate the shapes with three different idealized geometrical models, two of which are based on idealized models proposed previously [5, 18], and one of which is novel and based on a polynomial fit. We compare the PVS-to-artery area ratio and hydraulic resistance of each modeled shape to those of the segmentation and find that agreement is closest for the polynomial fit. We report detailed statistical distributions of various measures of PVS size, shape, and hydraulic resistance, based on the parameters in the idealized models, which will be useful in modeling and can serve as a baseline comparison for diseases in which PVS size and shape are biomarkers.

## Methods

### Animals

All procedures involving animals were in compliance with the experimental protocol approved by the University Committee on Animal Resources of the University of Rochester (Protocol No. 2011–023), certified by the Association for Assessment and Accreditation of Laboratory Animal Care. Efforts were taken to keep animal usage to a minimum. Male and female BPN/3J mice (Jackson Labs, JAX stock #006567) or *β*-Act-GFP mice (Jackson Labs, JAX stock #006567) on a C57BL/6 background 8–12 weeks of age were used for all the experiments. *β*-Actin-GFP mice show a widespread expression of enhanced green fluorescent protein (EGFP), with the exception of erythrocytes and hair ([29]). All experiments performed in this study were done on mice anesthetized with ketamine and xylazine (100 and 10 mg/kg, intraperitoneally). The depth of anesthesia was determined by the pedal reflex text. Once reflexes had cased, anesthetized mice were fixed in a stereotaxic frame for the surgical procedure, and body temperature was maintained at 37.5 °C with a rectal probe-controlled heated platform (Harvard Apparatus).

### Intracisternal injections and CSF tracers

Anesthetized mice were fixed in a stereotaxic frame. A 30-gauge needle was connected to a PE-10 tubing filled with artificial CSF (aCSF) and inserted into the cisterna magna as described previously [30]. Alexa Fluor647– or Alexa Fluor594–conjugated bovine serum albumin (BSA-647 or BSA-594, 66 kDa, Invitrogen) were diluted in artificial CSF at a concentration of 0.5% (w/v) and used as a fluorescent CSF tracer. For intracisternal injections, 10 *μ*l of CSF tracer was injected at a rate of 2 *μ*l/min over 5 min with a syringe pump (Harvard Apparatus).

### In vivo two-photon laser scanning microscopy

A cranial window was prepared over the right anterolateral parietal bone above the MCA vascular territory. The dura mater was left intact and, to prevent intracranial depressurization, the window was sealed with agarose (1% at 37 °C) and a glass coverslip (5 mm diameter). Two-photon imaging was performed using a resonant scanner Bergamo scope (Thorlabs) and a Chameleon Ultra II laser (Coherent) with a water-immersion 20× objective (1.0 NA, Olympus). Intravascular FITC-dextran (2000 kDa, 1%, Sigma-Aldrich), and intracisternal CSF tracers BSA-647 or BSA-594 were excited at 890 nm wavelength. Emission was filtered at 525, 607, and 647 nm. After the tracer reached the PVS of the cortical arteries, volumetric 3D images were acquired. The green channel captures the FITC–dextran in the vasculature, while the red channel and far-red channels capture the CSF tracer flowing in the perivascular space. Note that in the figures presented in this study, we have changed the colors of the channels to improve clarity (red for FITC Dextran tracer in the vasculature, blue for Alexa Fluor tracer in PVS, and green for the green fluorescent protein). Images obtained were 16 bits with spatial dimensions of 512 by 512 pixels. The image resolution in the transverse plane was 0.648 *μ*m/pixel, while the resolution for cortical depth was 1 *μ*m/pixel.

### Image Segmentation

To define the boundaries of the vessel and perivascular space, we segmented the images based on tracer intensity. Because the fluorescent signal intensity is attenuated as it passes through red blood cells and tissue, we used a depth-varying intensity threshold, classifying a region as part of the vessel (or PVS) when its intensity exceeded the threshold. For each vessel and PVS, we tried four different semi-automatic approaches to determine the depth-varying threshold: Mean + Standard Deviation, Noise Finder, Otsu’s method, and Edge Finder (described further in Additional File 1). The Edge Finder approach most correctly identified the vessel and PVS in the majority of the cases, and when it performed poorly, Mean + Standard Deviation was used as a substitute. Otsu’s method and Noise Finder were not used because they over-segmented and under-segmented the image respectively, as further described in Additional File 1. After segmenting images according to the threshold, we fine-tuned and compared the resulting segmentation to the original image. For each vessel and PVS, the segmentation was quantitatively validated by comparing a single plane of the 3D image segmentation with the segmentation of a single plane time-averaged time-series image (which has a considerably higher signal-to-noise ratio), as described in the validation section.

The Edge Finder approach determined the threshold at each depth based on the maximal gradient magnitude. Prior to segmentation, the image was smoothed with a 3D Gaussian filter (using the built-in MATLAB function “imgaussfilt3”) with a standard deviation of 2 voxels. At each depth, we identified edges (regions of the maximal gradient magnitude) using MATLAB’s “edge” command and set the threshold equal to the median intensity value on the edges. The resulting threshold did not always monotonically decrease with depth as we would expect, as shown in S1, Additional File 1 (dashed black line), so we refined the threshold by manually defining, based on the raw signal, a depth-varying threshold that is constant at shallow and deep depths and decreased linearly between the constant regions. Then we compared the resulting segmentation with the original intensity image and iteratively modified the refined threshold until the segmentation qualitatively agreed with the vessel/PVS location in the raw intensity images. The resulting threshold “Edge Finder” is shown in S1, Additional File 1 (green line). For the majority of the cases, the Edge Finder approach segmented the PVS and vessel well, but in a few select cases (Additional File 2 G, I, and J (PVS only)), it did not work well and the Mean + Standard Deviation approach was used instead.

The Mean + Standard Deviation approach set the threshold equal to the sum of the mean and standard deviation of all intensity values at each depth. The depth-varying threshold array was then modified so that the threshold at all depths shal-lower than the location of the maximum threshold was equal to the maximum.

The resulting segmentation often contained small exclaves. We removed all regions that were smaller than 5–10 pixels at each depth. We also removed all but the single largest connected region in the vessel segmentation and all but the two largest regions in the PVS segmentation. Once these disconnected regions were removed, all the holes in the segmentation were filled. We manually removed the vessels that branched off of the main vessel of interest.

In some cases, the segmentation of PVS and vessel overlapped by a few pixels. To correct this, the segmentation for both the PVS and vessel was compared to the intensity images in both transverse (*x*-*y*) and axial (*x*-*z* or *y*-*z*) planes to qualitatively determine which one matched most closely the original data. The most reliable of the two segmentations was used to subtract all the pixels from the other segmentation to remove the overlapping regions.

**Figure 2:**
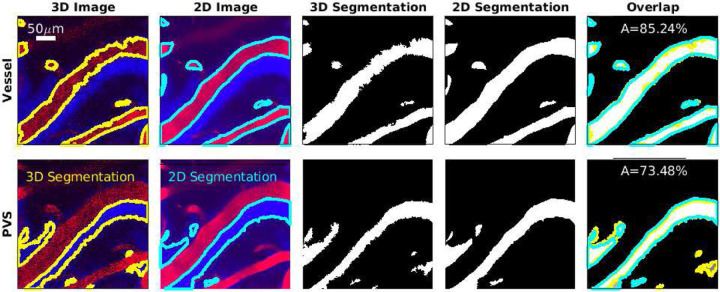
Comparing the 3D segmentation to a high-quality 2D segmentation. Images show vessels (top row) and PVSs (bottom row) at cortical depth 51 *μ*m. In the two leftmost columns, yellow and cyan indicate segmentation boundaries. In the rightmost column, regions present only in the 3D segmentation are dark gray with yellow boundaries, regions present only in the 2D segmentation are light gray with cyan boundaries, and regions present in both are white. The area overlap A is used to determine the segmentation quality. The 3D segmentation and 2D segmentation match over 70% which gives confidence that 3D segmentation is accurate.

To validate the 3D segmentation, we compared the segmentation at a single depth to the segmentation of a 2D image with a high signal-to-noise ratio, as shown in Figure 2. In the majority of cases, in addition to 3D images, we also acquired images at a single depth at 30 frames per second for several minutes, then averaged 1000 frames from the time series, which resulted in one high signal-to-noise image, which we segmented. To determine the depth of the 2D images, the 3D images were compared to the 2D time series to find the cortical depth that minimized the difference between the two. Once the depth was determined, we identified any translation and rotation of the field of view between the 2D time series and 3D images and aligned them. Additional details on the 2D-to-3D registration are described by Boster et al. [31]. Once the 2D and 3D image series were aligned, the segmentations were overlaid on top of each other, and the percentage of overlaid area compared to the total area for PVS and vessel segmentation was obtained by

(1) $$A_{\text{percentage}}=\frac{A_{2D}\cap A_{3D}}{A_{2D}\cup A_{3D}},$$

where *A*_*2D*_ is 2D segmentation and *A*_*3D*_ is 3D segmentation shown in Figure 2. To consider the segmentation successful, at least 70% of the area had to overlap in both PVS and vessel.

### Creating normal cross sections

**Figure 3:**
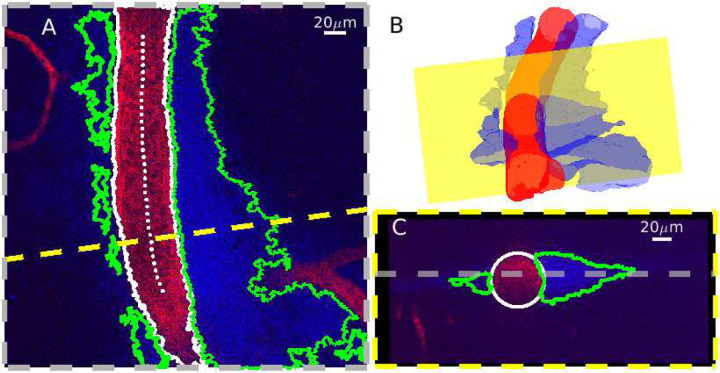
Normal slices obtained from the 3D segmentation. **A** Single image from an example z-stack (z=70 *μ*m) with the vessel centerline (dotted white), vessel segmentation boundaries (white), and PVS segmentation boundaries (green). The dashed yellow line indicates a cross section orthogonal to the vessel centerline. The vessel lumen appears red while the PVS lumen appears blue. **B** Rendering of the segmented vessel and PVS. The yellow plane corresponds to the dashed yellow line in (A). **C** The cross section is indicated in (A-B). The gray line marks the depth of the image in (A). We obtained cross sections of the 3D image that were normal to the vessel centerline.

In order to quantify PVS sizes and shapes, we sampled the original 3D image at cross sections normal to the vessel centerline. The segmented volumes were first re-sampled to make their resolution isotropic. We estimated the vessel centerline using MATLAB’s “bwskel” function, then manually removed erroneous points. We fit a second-order polynomial to the points in the transverse (*XY*) and axial (*XZ* or *YZ*, depending on the vessel orientation) planes, and obtained a vector normal to the vessel centerline at each vessel center point by crossing vectors that are perpendicular to the polynomial fits. The MATLAB function “obliqueslice” was then used to create cross-sectional slices using these normal vectors and their center points as shown in Figure 3. The slices were then shifted so the center point of the vessel was located in the center of the image. Cross sections located near the edge of the acquisition volume often did not capture the full width of the PVS. We excluded cross sections where the distance from the center of the vessel to the edge of the imaging plane was less than the median width of the PVS divided by 2.5. This resulted in between 54 and 451 different cross sections for each of the 14 PVS segments that were included in the subsequent analysis. The average distance between normal planes was approximately 0.8 *μ*m. Each cross section was analyzed separately, but in order to create a representative image of the cross section from each location, we averaged all of the images along the vessel centerline, as shown in Additional File 2.

### Idealized geometries

**Figure 4:**
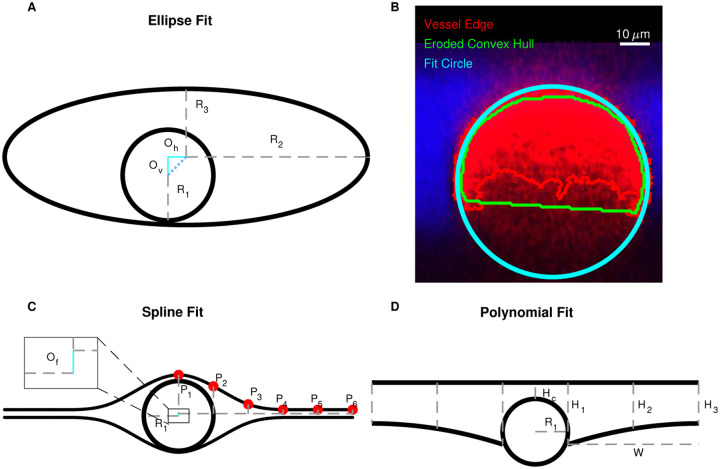
We fit three different idealized geometries to the segmented PVS, and we fit a circle to the vessel. **A, C**, and **D** Three idealized PVS geometries: spline, polynomial, and ellipse fits. The spline fit is defined by vessel radius *R*_1_, offset *O*_*f*_, and points *P*_1_ – *P*_6_, whose locations are specified in Table 1. The polynomial fit is defined by vessel radius *R*_1_, width W from the vessel to the end of the PVS, height *H*_C_ from the top of the vessel top of the PVS, height *H*_1_ of the PVS next to the vessel, height *H*_2_ of the PVS midway between the vessel and the end, and height *H*_3_ of the PVS at the point furthest from the vessel. The ellipse fit is defined by vessel radius *R*_1_, major axis *R*_2_, minor axis *R*_3_, horizontal offset *O*_h_, and vertical offset *O*_v_. We fit each of these idealized shapes to the PVS and vessel segmentation to determine which best characterized the PVS. **D** A cross section of a vessel and an outline of its segmentation. We fit a circle to points located on the edge of the segmentation. Because image quality degraded with depth, the segmentation on the bottom of the vessel has greater uncertainty. Edge points that were inside an eroded convex hull of the segmentation were excluded from the fit.

We characterized PVS shapes by fitting the segmented PVSs in each of the normal cross sections to three different idealized shapes, depicted in Figure 4: spline, polynomial, and ellipse fits. We approximated the vessel as a circle and used the same vessel fit for all three fits (Figure 4).

Red blood cells attenuate the fluorescent signal more aggressively than other tissues, reducing the signal-to-noise ratio in the bottom portion of the vessel and resulting in higher uncertainty in the segmentation. To compensate, we fit a circle to points located on the edge of the segmentation, excluding the edge points that were located inside the eroded convex hull of the segmentation which effectively eliminated the uncertain edge points on the bottom of the vessel, as shown in Figure 4.

The ellipse fit is based on the work of Tithof et al. [18], who suggested idealizing the PVS and the vessel using a fitting ellipse and circle, respectively, with the same centroids and the same second central moment as their corresponding segmentation. However, we used the approach described above for the vessel fit because it worked better (based on visual inspection comparing the resulting segmentations with the original intensity image) than the second-moment approach because of the uncertainty associated with segmentation on the bottom portion of the vessel.

The spline fit is inspired by an idealized shape proposed by Vinje et al. [5]. They defined the shape with spline curves. We defined six control points that are connected by a spline, approximately based on their description. The locations of the control points are listed in Table 1 and shown in Figure 4. We defined the points in terms of the vessel radius, so the geometry scales with vessel size only, and not PVS size or shape, in contrast to the ellipse and polynomial fits. *R*_1_/4.5 is the minimum width the PVS tapers to at either end of the fit. The splines are then mirrored across horizontal and vertical axes to create the PVS in Figure 4. The parameter *O*_*f*_ = 0.0667*R*_1_ indicates how far below the midpoint of the PVS the vessel center is located.

In the polynomial fit, we idealize the top of the PVS as a line and the bottom of the PVS as two second-order polynomials, as depicted in Figure 4. We find the coefficients for the line from a least-squares fit of points located along the top edge of the segmented PVS. We find the coefficients for the second-order polynomials from a least-squares fit of points located on the bottom edge of the segmented PVS.

### Hydraulic resistance

We calculated the resistance per unit length for flow through straight channels with cross sections corresponding to the PVS segmentation and the three fits for each normal cross section. We assume a fully developed Poiseuille flow, which results in a unidirectional flow governed by Poisson’s equation. We solved Poisson’s equation numerically with MATLAB’s “solvepde”. For each cross section, we first eroded the PVS segmentation or idealized fit by one pixel then found the location of the segmented PVS boundary using MATLAB’s “bwboundaries”. We used the “polyshape” and “geometryFromMesh” commands to create a model that was discretized using MATLAB’s “generateMesh” command, creating a triangular mesh. We refined the mesh until the element size was small enough that the error in resistance for a circle of the same area was less than 1%. We used the viscosity of water at 36 °C, 7.058 ×10^−4^ Pa · s. The hydraulic resistance is independent of the pressure gradient, but for the sake of determining a corresponding velocity profile, we used a pressure gradient of 500 Pa/m [31].

### Defining the distribution peak and excluding outliers

We report the peak of the distribution to describe the most common value, as opposed to the mode or mean, because the distributions shown are of continuous values that are not normally distributed. To determine the peak, we use 20 evenly spaced bins, and the peak value is the bin center with the largest probability density. We also report the 25^th^, 50^th^, and 75^th^ percentiles and interquartile range (IQR) in Table 2 to indicate the range of typical values, rather than the standard deviation, since the values are typically not normally distributed.

**Figure 5:**
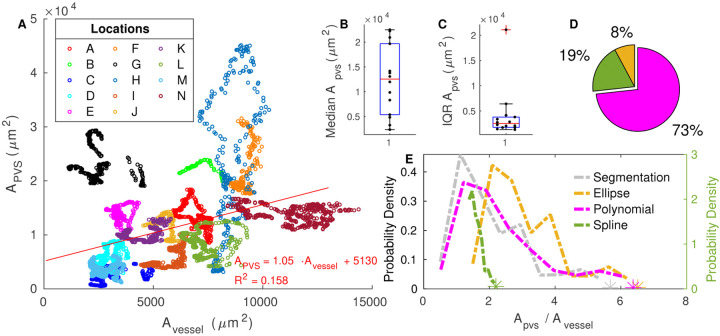
Variation in PVS area. **A** Area of the perivascular space and area of the vessel for each cross section in the 14 (A-N) different segments. The least-squares fit of the PVS area as a function of the vessel area is shown in red. The PVS area only approximately scales with the vessel area. **B, C** the median and interquartile range of the segmented PVS area from each of the 14 different PVS segments (A-N). Intrasegment variability is much lower than intersegment variability. **D** Share of segmented cross sections whose area ratios are best predicted by each model. Colors correspond to the legend in (E). **E** The distributions of the PVS-to-vessel area ratio across all cross sections from all vessels, based on the original segmented regions and the various fits. The spline fit is plotted on the right axis because its area ratio is nearly constant, resulting in a larger probability density than the other fits. The area ratio of the annulus model is chosen to match each segmentation exactly and thus is not displayed. The polynomial fit distribution most closely matches that of the segmentation and is the best match for the majority of cross sections.

The distributions of hydraulic resistance and area ratio often contained large outliers that skewed the distributions. The outliers occurred when the automated segmentation and fits did not work well. In order to more clearly show the trends in the distributions, in Figures 5, 8, 9, Additional File 3, Additional File 5 and Additional File 6 we do not show the outliers, but instead plot an asterisk at the point where the outliers begin. We define as outliers any points that are more than 1.5 times the interquartile range above the 75^th^ percentile or below the 25^th^ percentile.

## Results

### Area ratio

The PVS-to-vessel area ratio is often reported as a convenient measure of PVS size, but we found that the PVS area scales only approximately with vessel area. We show the segmented PVS and vessel area for each cross section in Figure 5A. The area of the PVS generally increases with the area of the vessel, but the trend is only approximate, as evidenced by the low R^2^ value of the fit line (0.158). The slope of the fit line is 1.05, (95% confidence interval 0.973, 1.12). We show the distribution of segmented PVS-to-vessel ratios in Figure 5E. There is considerable variation in the area ratio, and the interquartile range of the segmented area ratio is 1.79 (Table 2), a value on the same order as the median value of 1.88, meaning that the area ratio varies by more than 100%. The peak value of 1.12 agrees well with the slope of 1.05 from the fit line in Figure 5 and literature estimates of 1.26 [2] and 1.4 [28]. Clearly, the PVS area ratio has considerable variation, and additional parameters are needed to more accurately quantify PVS size than the area ratio alone.

From Figure 5, it is clear that the variation in PVS area is much larger between segments of PVS than along the length of a single PVS segment. We quantified this difference by plotting the median PVS area from each segment and the interquartile range of each segment in the upper right of Figure 5. The same is also true for the area ratio (Additional File 3). The peak of the area ratio distribution ranges from 0.6 to 11.43 across the different locations. Although their area ratio varies along the length of each vessel, in all but one case, this variation occurs within 30% of the peak, showing inter-segment variation is the main contributor to the variation in area ratio.

We used different fits to quantify the size and shape of the PVS. To determine which fit best represents the PVS, we compare the area ratio and hydraulic resistance of the original segmentation with those of each of the fits. The distribution of the PVS-to-vessel area ratio in segmentations is matched most closely by the polynomial fit (Figure 5E), and the value of the area ratio in segmentations is matched most closely by the polynomial fit in 73% of cases (Figure 5D). The peak of the area ratio distribution for the polynomial fit is 1.21, similar to the peak of the segmentation distribution of 1.12. The distribution for the ellipse fit is also similar to that of the segmentation. However, the ellipse fit gives the closest match in only 8% of cases and has a significantly higher peak of 2.08 because it does not narrow at the ends. The spline fit gives the closest match in 19% of the cases and has a peak of 1.48, but the distribution is very different from that of the segmentation and the other fits: the area ratio has very little variation because, by definition, the PVS size scales with the vessel size, resulting in a nearly constant area ratio.

### Hydraulic Resistance

The hydraulic resistance (per unit length) is defined as:

(2) $$R\equiv \frac{\partial P/\partial n}{Q},$$

where *Q* is the volume flow rate and ∂*P*/∂*n* is the pressure gradient in the direction of flow, and it is a key parameter for describing how the shape affects the flow. To determine how the resistance of the idealized shapes compares with the segmented PVS, we calculated the hydraulic resistance for flow through straight channels with cross sections that match the shapes of the PVS segmentation and fits at each of the normal cross sections. This results in a uni-directional, fully developed flow. In reality, the shape varies along the length of the channel resulting in non-axial flow components, but they are small compared to flow in the axial direction. Boster et al. showed that assuming a unidirectional, fully developed flow in a straight cross section resulted in errors on the order of 10% for a realistic PVS geometry [31]. Therefore, calculating the hydraulic resistance for fully developed straight channel flow is a convenient simplification that provides insight into how different shapes affect the resistance.

**Figure 6:**
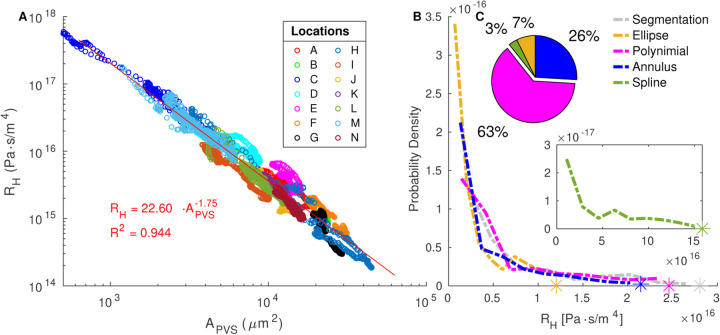
Variation in hydraulic resistance. **A** Hydraulic resistance and area of the perivascular space for the segmentation at each cross-section. The hydraulic resistance approximately scales with PVS area. **B** The distributions of hydraulic resistance per unit length across all cross sections from all vessels based on the original segmented regions and the various fits. The spline fit is plotted separately (lower inset) because its resistance is larger than the other fits. **C, Upper inset** Share of segmented cross sections whose hydraulic resistances are best predicted by each model. The polynomial fit distribution most closely matches that of the segmentation, and the polynomial fit was the best match for the majority of cross sections.

Figure 6A displays the hydraulic resistance of each segmented cross section as a function of the segmented PVS area. The hydraulic resistance can be reasonably predicted with a power-law fit as a function of the PVS area alone (R^2^ = 0.944). The exponent in the power law fit is −1.75 (95% confidence interval −1.763, −1.737), similar to the value of −2 that is true for a circle, ellipse, or any shape as long as only the size scales without otherwise altering the shape.

The hydraulic resistance of each cross-section from each of the 14 different locations (A-N) is shown in Figure 6B, and it is evident that the variation between locations is much larger than that along the PVS at each location. The peak of hydraulic resistance distributions ranges from 3.23×10^14^ to 1.98×10^16^ across different locations, whereas, in 65% of the vessels, all the variation occurs within 37% of the peak of the distribution (Additional File 5), showing that inter-segment variation is much larger than intra-segment variation.

Figure 5B shows that the distribution of hydraulic resistances for the polynomial fit matches that of the segmentation most closely, followed by the ellipse fit. We also plot the resistance of an optimal concentric elliptical annulus or the concentric elliptical annulus that results in the lowest resistance for a given area ratio *K*, which can be approximated as

(3) $$R_{H}={\left(\frac{\mu \cdot 6.67\cdot K^{-1.96}}{R_{1}}\right)}^{4}$$

where *R*_1_ is the radius of the vessel in the optimal elliptical annulus, and *μ* is the viscosity, as described by Tithof et al. [18]. We used the area ratio K from the segmentation, rather than the ellipse fit, since the ellipse fit tended to overestimate the area ratio. The distribution of resistances for the optimal elliptical annulus is very similar to that of the ellipse fit, which supports the finding of Tithof et al. that the shape of a PVS, as defined by the ellipse fit, is very close to the optimal ellipse. This fact is a convenient feature of the ellipse fit: the hydraulic resistance can be reasonably approximated with knowledge of the area ratio alone. Though both the ellipse fit and annulus tend to underestimate the resistance of the PVS, the convenience of being able to calculate the resistance with such a simple analytical equation may make the trade-o in accuracy worthwhile in some situations.

The resistance for the spline fit is considerably larger than that of the segmentation (distribution peak is 5 times larger), so the distribution is plotted separately, in the inset of Figure 5B. The larger resistance is a result of the shape of the fit. Despite having a larger area ratio than the segmentation and polynomial fit, the resistance is higher because of the way the PVS rapidly narrows as it moves away from the vessel, illustrating the importance of modeling not just the correct PVS area, but a reasonable approximation of the PVS shape as well.

**Figure 7:**
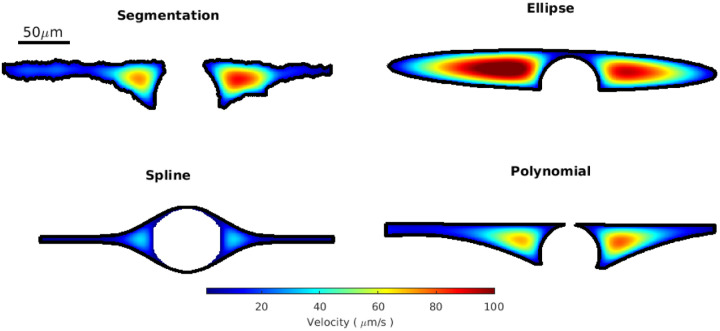
Simulated velocity profiles in the PVS for one representative cross section. In this example case, the velocity profile for the polynomial fit is similar to the velocity profile in the segmentation, whereas the ellipse fit results in much higher velocities, and the spline fit results in much lower velocities.

To further emphasize this point, in Figure 7 we show the velocity profiles for each fit in a representative cross section. The spline fit has the slowest speeds because the space adjacent to the vessel is narrower than the other fits. This highlights the importance of considering not just the size but also the shape of the PVS. The velocity profiles for the ellipse and polynomial fit are more similar to that of the segmentation. These trends are generally consistent in all cross sections, as reflected in the hydraulic resistance distributions. The fastest speeds for all of the cross sections in Figure 7 occur close to the vessel where the space is widest. Because of the way the PVS tapers, the speed in the regions far from the vessel is much slower, and those regions could probably be excluded without significantly impacting the predicted mass and momentum transport rates volume flow rates (e.g., volume flow rate, mass flux).

### Characteristics of the typical PVS shape

**Figure 8:**
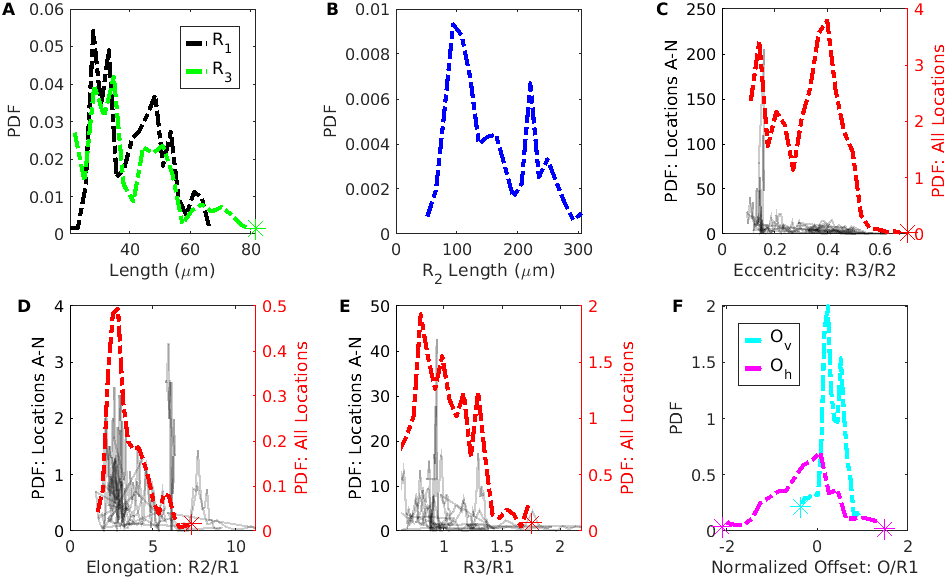
Distributions of ellipse fit parameters, as defined in Figure 4. The PVS shape is typically narrow (*R*_3_/*R*_2_ ~ 0.4) and elongated (*R*_2_/*R*_1_ ~ 2.9) with the vessel slightly offset down from the PVS center.

**Figure 9:**
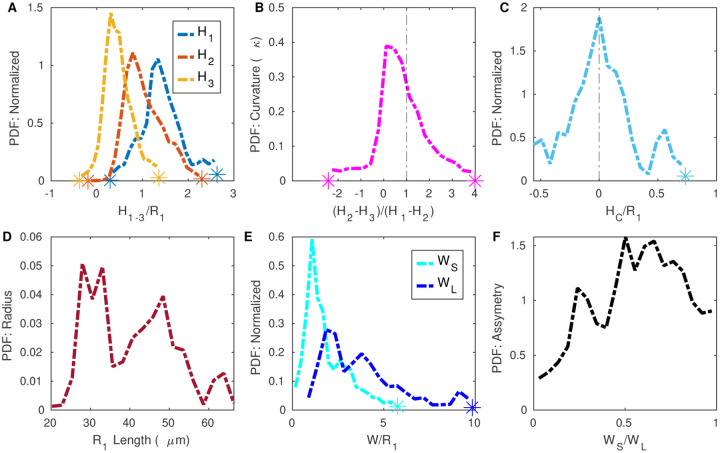
Distributions of polynomial fit parameters, as defined in Figure 4. W_s_ and W_L_ are the widths of the short and long sides of the perivascular space, respectively. The height of the perivascular space is the greatest next to the vessel.

To quantify the PVS shape and size, we report the distributions of the ellipse and polynomial fit parameters (Figures 8 and 9), and various statistics for the polynomial fit (Table 3). To accurately model mass and momentum transport, it is essential to know both the area and shape of the PVS, as demonstrated by the significant difference in hydraulic resistance between the spline fit and segmentation, despite having a similar area. The area ratio has previously been reported as greater than 1 [2, 28], but the shape of the PVS has not been rigorously quantified previously. Here we describe some general characteristics of the PVS shape derived from the distributions of parameters in the polynomial and ellipse fits.

The parameter distributions from both the polynomial and ellipse fits show that the shape of the PVS is comprised of two mostly or entirely disconnected regions on either side of the vessel (Figures 8A, E, and 9C). For the ellipse fit, the distribution of *R*_1_, the vessel radius, is similar to that of *R*_3_, the minor axis length (distribution peaks at 28 *μ*m and 35 *μ*m, respectively, see Figure 8A), and the ratio between them (*R*_3_/*R*_1_) is generally close to 1 (distribution peaks at 0.80, Figure 8E). Similarly, in the polynomial fit the parameter *H*_C_ is the distance between the top of the polynomial fit and the top of the vessel (as illustrated in Figure 4D), with values greater than zero indicating left and right sides of the PVS are connected, and values less than zero indicated that the PVS is disconnected. The distribution peaks at zero (Figure 9C), suggesting that the top of the vessel is most often very nearly aligned with the top of the PVS and that the PVS is often separated into two lobes. Even when the two sides are connected, the channel connecting them is quite narrow, usually less than 0.5*R*_1_. The connection is narrow (relative to the height of the PVS at *H*_1_ and *H*_2_), suggesting that the channel, when present, has little effeect on the hydraulic resistance, making its presence or absence inconsequential for the hydraulic resistance.

The PVS is typically elongated, with a width approximately twice the vessel radius. The ratio of the major axis to vessel radius of the ellipse fit (*R*_2_/*R*_1_) distribution peaks at 2.89 (Figure 8D). The major axis of the ellipse fit represents the distance from the PVS center and so, it includes the vessel radius. Thus, the width of the PVS is approximately twice the vessel radius. Similarly, in the polynomial fit, the ratio of PVS width to vessel radius (*W*_S_/*R*_1_) peaks at 1.85 (Figure 9E), suggesting that the PVS is twice as wide as the vessel radius.

The PVS is typically eccentric, with a height much smaller than the width. The ratio of the minor axis to the major axis (*R*_3_/*R*_2_) peaks at 0.398 (Figure 8C) meaning that typically, the minor axis is more than two times smaller than the major axis.

The PVS is often asymmetric in that the vessel is typically not located in either the vertical or horizontal center of the PVS. The asymmetry is apparent from the distributions of parameters that describe both the polynomial and ellipse fits. *O*_v_ is and vertical off set between the center of the ellipse and the center of the vessel, and the distribution peaks at 0.24 (Figure 8F), indicating that the vessel is most frequently slightly lower than the center of the ellipse. *O*_h_ is the horizontal off set between the center of the ellipse and the center of the vessel and is centered around zero, indicating no preference in off set for one side relative to the other. However, the distribution for the horizontal off set is much wider than that of the vertical off set (Figure 8F), indicating that the vessel is often shifted dramatically from one side to the left or right of the PVS center. This horizontal shift is characterized more clearly in the polynomial fit, where the PVS width W is the distance from the edge of the vessel to PVS edge. For each cross-section, there is a width measurement from the left and right side of the PVS. Since there was no statistically significant difference between the left and right sides, we report the distribution of the short (*W*_S_) and long (*W*_L_) sides of each cross section. The short side is significantly shorter than the long side (distribution peaks of 1.1 and 1.9, respectively, Figure 9E) and for the ratio between them, *W*_S_/*W*_L_, the distribution peaks at approximately 0.5, indicating that the two PVS sides are typically asymmetric, a feature that results in reduced hydraulic resistance compared to a symmetric shape of the same area ([18]).

The PVS narrows dramatically further from the vessel. One difference between the polynomial and ellipse fits is highlighted in Figure 9A, where the distributions of heights of the PVS, *H*_1_, *H*_2_, and *H*_3_, peak at 1.34, 0.792, and 0.307, respectively, indicating that the PVS is larger closer to the vessel and smaller at the ends of the PVS. *H*_1_, the height of the PVS close to the vessel, is 4 times larger than *H*_3_, the height of the PVS far from the vessel, indicating that the PVS is typically thicker close to the vessel and thinner further away from the vessel and suggesting a dramatic change in height along the length of the PVS, a feature not captured by the ellipse fit.

The PVS is often concave. The ratio of the change in heights, (*H*_2_−*H*_3_)/(*H*1−*H*2) shown in Figure 9F describes the curvature of the bottom edge of the PVS, with values greater than one indicating the PVS is convex, and values less than one indicating the PVS is concave. The peak of the curvature distribution is 0.108, showing that the PVS is most often concave, rather than convex, as is required by the ellipse fit. This concavity has implications for the resistance to flow, since the resistance would be larger for a concave shape of the same area than a convex shape, and is a large part of why the ellipse, which is convex, has a smaller resistance than the segmentation.

### Optimal model of the PVS

Despite having a similar peak in area ratio, the spline fit has a much higher hydraulic resistance, which we attribute to the way the PVS rapidly narrows, creating a smaller space adjacent to the vessel. The narrow space results in slower speeds (Figure 7) and considerably higher hydraulic resistances (Figure 6B). Because of the way spline fit is defined only with respect to vessel size, there is very little variation in the area ratio (Figure 5 and Additional File 3). While the area ratio can be a useful parameter, it is clear from Figure 5 that the size of the PVS is not a function of the adjacent vessel’s size alone.

The ellipse fit has a lower hydraulic resistance than the segmentation, as a result of the larger area ratio and convex shape. The hydraulic resistance of the optimal annulus is more similar to that of the segmentation than the ellipse fit. This is because it is based on the area ratio of the segmentation at each cross section, while the ellipse is based on the second central moment of the PVS which overestimates the area ratio (Figure 5E). In other words, for the sake of calculating the resistance, if assuming an annulus, it is preferable to assume an optimal shape and use a measured area ratio than base the shape on the second central moment. The ellipse fit also assumes the PVS curvature to be convex, which results in a smaller hydraulic resistance for the same area. These features explain why the ellipse fit results in lower hydraulic resistance than the segmentation.

The polynomial fit results in area ratios and hydraulic resistances closest to those of the segmentation, suggesting that it captures the shape of the PVS best. The polynomial fit allows the PVS to narrow further from the vessel and to be concave, both of which contribute to its ability to capture the PVS shape. If we consider the complexity of the fits and how flexible they are across different cross sections, the polynomial fit is defined in a way that can most closely fit the variations as seen in Additional File 1 across the 14 locations. Though the shape is not as simple as the other fits, it can be fully described with only a few more parameters.

**Figure 10:**
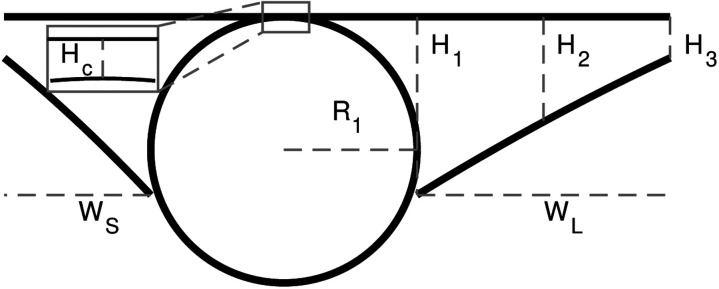
Quintessential Polynomial fit geometry. The parameter values were determined based on the most common parameters (defined as the peak of the distribution) across all analyzed cross sections.

The variation in PVS shape is evident from the wide distributions of parameter values in Figure 9 and Additional File 2, but for modeling purposes, it is practical to define a single “idealized” or quintessential PVS shape that is representative of the most common PVS shape. We based the representative PVS geometry on the polynomial fit as it more accurately captures the PVS shape compared to the other fits that we explored, and we determine the parameter values based on the most common or peak values from the distributions. The representative fit is shown in Figure 10, and the most common, 25^th^, 50^th^, and 75^th^ percentile parameter values are listed in Table 3.

## Discussion

Filled with flowing CSF, PVSs constitute primary pathways for fluid and solute transport, which is strongly affected by PVS size and shape. In this study, we have characterized the size and shape of thousands of different PVS cross sections in an effort to better define the PVS geometry and aid model development. Our PVS-to-vessel area ratio measurements are consistent with those previously reported. Schain et al. [2] and Mestre et al. [28] reported area ratios of 1.26 and 1.4, similar to our peak of 1.12 and a median of 1.88. The PVS-to-vessel area ratio approximately scales with arterial size, as shown in Figure 5 and previously reported [27, 32]. However, as the wide distribution in Figure 5A and the poor fit in Figure 5 demonstrate, the scaling is only approximate and the area ratio varies considerably.

We approximated the cross-sectional shape of the vessel as a circle and found that the peak of the vessel radius distribution is 28 *μ*m (Fig 8A and 9C). Schain et al. [2] reported and range of 6 to 11 *μ*m for the radius of the arteries and arterioles. The measurements we report all came from locations located approximately 4 to 7 bifurcations downstream from the main middle cerebral artery. Since their measurements included arterioles as well as venules, it is possible that their measurements were further downstream, which could potentially explain the difference. Mestre et al. [28] measured the pial radius to be 23 *μ*m, similar to the values we report.

Though the shape of pial PVSs has not been quantified previously, quantitative measurements agree with the qualitative descriptions in the literature. Mestre et al. [28] and Tithof et al. [18] described the PVS as non-connecting with two disjoint compartments forming on each side of the pial arteries. An elliptical shape with two disjoint PVSs was also assumed by Kedarasetti et al. [33]. In agreement with our measurement that the PVS width is approximately twice the vessel radius, Mestre et al. [28] reported the PVS width in a single plane to be about the same as the vessel width (twice the vessel radius). Rudie et al. [4] report that the PVS is generally a well-defined oval, rounded or tubular structure with smooth margins. Tithof et al. reported that the PVS outer boundaries are often oblate and that the PVS around the pial artery is an annular region, elongated in the direction along the skull [18]. They also noted that PVS eccentricity occurs and noted that eccentricity reduces hydraulic resistance because fluid is further from the wall, allowing for higher velocity for a given driving pressure.

The hydraulic resistance dictates the speed of CSF flow in the PVS, determining the rate at which biological waste like proteins and dead white blood cells is removed [34]. Resistance is a key parameter for large-scale models of cerebral CSF flow [19, 21, 35, 36] and has previously been estimated by assuming a uniform PVS geometry. Here, we report the distribution of hydraulic resistances for thousands of PVS cross sections, with its peak at 1.73×10^15^ Pa· s/m^4^. In their hydraulic network model, Tithof et al. [35] used a hydraulic resistance of 8.7×10^15^ Pa· s/m^4^ for pial vessels, assuming an optimal concentric elliptical annulus with an area ratio of 1.4 and vessel radius of 23 *μ*m, based on measurements reported by Mestre et al. [28] and Tithof et al. [18]. This hydraulic resistance is larger than our segmentation resistance, suggesting that the value they used for pial PVSs overestimated the hydraulic resistance. However, since the vast majority of the resistance in their model comes from other regions of the network (e.g. the porous penetrating and capillary PVSs and parenchyma), this overestimation of pial PVS resistance is not expected to alter their conclusions. The concentric circular annulus assumed by Faghih and Sharp results in a resistance of 1.4×10^16^ Pa· s/m^4^ for a vessel radius of 28 *μ*m [19], suggesting that their model overestimates the hydraulic resistance. In future studies, PVS resistance can be estimated from PVS size using the empirical relation we described above.

PVSs play a crucial role in clearing interstitial solutes such as *β*-amyloid from the brain [1, 4, 8, 28, 37]. PVS shape affects the hydraulic resistance, velocity profile, and transport rate of the CSF. Vinje et al. showed the importance of PVS shape [5]. They used numerical simulations to show that PVS velocity is linearly proportional to PVS width. In contrast, the mean velocity of a pressure-driven flow in a cylindrical vessel is linearly proportional to the square of the vessel radius. They also observed that differences in arterial and venous PVS shape would lead to differences in velocity and transport rate for a given pressure gradient [5]. The simple, parameterized fits of the PVS shape with the parameter distributions we report could be used to determine how various aspects of the shape affect fluid and mass transport.

Ballerini et al. [22] show that PVS size and shape correlate strongly with white matter hyperintensities on MRI scans of human subjects. Enlarged PVSs are also associated with small vessel disease and hypertension [38, 39]. Thus, it is important to benchmark the size and shape of PVSs in non-pathological scenarios, as we did here with detailed statistical distributions. Furthermore, the parameterized fits we describe could be used to more completely quantify the PVS shape, which may increase the sensitivity and specificity of using PVS metrics to detect PVS-related pathologies. The parameters could potentially be used as biomarkers of disease. Being able to more accurately quantify PVS size and shape could improve our understanding of and ability to detect how PVSs are altered in pathological conditions.

There are limitations to our study. Segmentation is inherently challenging due to the lack of ground truth data for comparison. We partially validated our segmentations by comparing them with a high signal-to-noise-ratio composite time-series image in a 2D transverse plane, which provided a quantitative method to evaluate the segmentation, but only at a single depth. After the initial binarization of the images, some of the post-processing steps, especially manual correction, were applied on a case-by-case basis, introducing a degree of subjectivity to the segmentation, and resulting in another source of uncertainty. In the future, ground truth segmentation results, either from a phantom or simulated data, could be helpful in evaluating the accuracy of the segmentation process.

In addition, our analysis is limited to perivascular spaces adjacent to pial arteries in the territory of the MCA and does not describe penetrating PVSs or venous PVSs, which are expected to have considerably different sizes and shapes. Characterizing their shapes may be simpler, because studies to date suggest that they are essentially cylindrical, with vessels off set to one side [18]. The permeability of penetrating PVSs, however, is di cult to quantify but strongly influences model predictions [36]. Our analysis does not yet extend to the complicated shapes of PVSs around vessel bifurcations. Anecdotally we observed that PVSs were larger around bifurcations, consistent with prior anecdotal observations [2]. This phenomenon could partially explain the large variation in the area ratio we report. Future work could characterize PVS shapes near bifurcations in greater detail.

## Conclusion

We report the size and shape of pial PVSs from 3D two-photon images of 14 locations in 9 different wild-type mice. We segmented the PVS and vessel using a semi-automatic process, allowing us to quantify the size and shape of the PVS at thousands of cross sections. We show that the PVS area scales only approximately with vessel area, meaning there is considerable variation in the PVS-to-vessel area ratio. We calculate the hydraulic resistance per unit length in the segmented PVS cross sections and show that it can be reasonably approximated as a function of the PVS area. Three idealized PVS geometries (ellipse, spline, and polynomial fits) were evaluated on how closely they captured the size and shape of the segmented shape. The polynomial fit matched the area ratios and hydraulic resistances of the segmented shapes most closely. The peak in the PVS-to-vessel area ratio distribution is 1.12 and 1.21 for the segmentation and polynomial fit respectively, while the hydraulic resistance distribution peaks at 1.73×10^15^ and 1.44×10^15^ Pa ·s/m^4^ for the segmentation and polynomial fits, respectively. Idealized PVS shapes are convenient for the purposes of modeling and better understanding how changes in the shape affect mass and momentum transport. They allow us to quantify various morphological aspects of the shape (e.g., vessel off set from the PVS center) and explore how changing the shape affects resistance and velocity distribution. We construct a representative shape based on the polynomial fit and provide detailed distributions of the parameters so that variations from the representative shape can be quantified. Since pial arterial PVSs are the entryway for the CSF flow into the brain, accurately quantifying their size and shape is critical to developing effective fluid dynamics models of the cerebral CSF flow and further understanding the clearance of biological waste. This work will lead to more accurate models of pial PVSs and can serve as a benchmark for characterizing PVS size and shape in non-pathological scenarios.

## Supplementary Material

Additional File 1 Image Segmentation Full Description
Detailed description of the process used to segment the vessel and PVS.

Additional File 2 Intensity Images
Averages along the vessel center-line of all of the cross-sectional images at each of the 14 locations. Each panel (A-N) corresponds to a different PVS segment.

Additional File 3 Segmentation Fits
Segmentations and fits of the average images shown in Additional File 2, with vessel radius *r* indicated. The polynomial fit matched the PVS segmentation most closely in most cases

Additional File 4 PVS-to-vessel Area Ratio for 14 Locations
The distributions of PVS-to-vessel area ratio for each of 14 locations in 9 mice. Distributions for spline fits are narrower than others and are plotted on the right axes. In most cases, the polynomial fit most closely matches the segmentation in terms of area ratio distributions.

Additional File 5 Hydraulic Resistance for 14 Locations
The distributions of hydraulic resistance per unit length for each of the 14 locations. The spline fit resistance is always larger than that of the segmentation and other fits and in several cases is not visible because it extends beyond the range of the plots. (Additional File 6 repeats these plots, with the ranges adjusted so the spline fit distributions are visible.) In most cases, the polynomial fit most closely matches the segmentation in terms of resistance.

Additional File 6 Spline Fit Hydraulic Resistance for 14 Locations
The distributions of spline hydraulic resistance per unit length for each of the 14 locations.

## Figures and Tables

**Table 1:** Horizontal location of the control points defined with respect to the vessel horizontal center. The vertical location of the control points with respect to the vertical midpoint of the PVS (shown with a horizontal dashed line in Figure 4).

Control Points	P1	P2	P3	P4	P5	P6
**Horizontal Location**	0*R*_1_	*R*_1_	2*R*_1_	3*R*_1_	4*R*_1_	5*R*_1_
**Vertical Location Top**	1.1111*R*_1_	0.85*R*_1_	0.2667*R*_1_	0.1111*R*_1_	0.1111*R*_1_	0.1111*R*_1_

**Table 2:** Area ratio and hydraulic resistance for the fits and segmentation.

Area Ratio	Segmentation	Ellipse	Polynomial	Spline	
Peak	1.12	2.08	1.21	1.48	
25^th^ percentile	1.21	2.26	1.45	1.45	
50^th^ percentile	1.88	2.89	2.15	1.58	
75^th^ percentile	3.00	3.96	3.42	1.75	
IQR	1.79	1.70	1.97	0.3	
Hydraulic Resistance (Pa · s/m^4^)	Segmentation	Ellipse	Polynomial	Spline	Annulus
Peak	1.73×10^15^	7.06×10^14^	1.44×10^15^	1.07×10^16^	1.32×10^15^
25^th^ percentile	1.87×10^15^	7.02×10^15^	1.38×10^15^	1.21×10^15^	1.36×10^15^
50^th^ percentile	3.84×10^15^	1.55×10^15^	3.55×10^15^	2.58×10^15^	2.49×10^15^
75^th^ percentile	1.23×10^15^	5.23×10^15^	1.07×10^15^	7.07×10^15^	9.4×10^15^
IQR	1.04×10^15^	4.53×10^15^	9.32×10^15^	5.86×10^15^	8.04×10^15^

**Table 3:** Parameters from the polynomial fit for all cross sections.

Parameters (μm)	*H*_1_	*H*_2_	*H*_3_	*H*_*C*_	*W*_*S*_	*W*_*L*_	*R*_1_
**Peak value**	57.7	29.3	15.4	0.0359	27.9	34.1	64.9
**25**^**th**^ **percentile**	39.8	26.9	10.3	−4.84	31.4	40.0	88.0
**50**^**th**^ **percentile**	59.0	36.7	17.5	0.858	40.2	67.0	139.0
**75**^**th**^ **percentile**	75.6	56.5	27.9	10.2	48.5	125	234
Parameters	*H*_1_/*R*_1_	*H*_2_/*R*_1_	*H*_3_/*R*_1_	*H*_*C*_/*R*_1_	*W*_*S*_/*R*_1_	*W*_*L*_/*R*_1_	*W*_*S*_/*W*_*L*_
**Peak value**	1.34	0.792	0.307	0.000689	1.08	1.85	0.502
**25**^**th**^ **percentile**	1.17	0.743	0.273	−0.141	1.04	2.16	0.386
**50**^**th**^ **percentile**	1.41	0.987	0.455	0.0220	1.60	3.53	0.589
**75**^**th**^ **percentile**	1.76	1.37	0.707	0.209	2.93	5.23	0.762

## Data Availability

The datasets used and analyzed during the current study are available from the corresponding author upon reasonable request.

## Abbreviations

aCSF: artificial cerebrospinal fluid
BSA: bovine serum albumin
CSF: cerebrospinal fluid
FITC: fluorescein isothiocyanate dextran
MCA: middle cerebral artery
PVS: perivascular space
