# Supplementary material for: Sizes and Shapes of Perivascular Spaces Surrounding Murine Pial Arteries
Source: Res Sq. 2023 Feb 17:rs.3.rs-2587250. Preprint. [Version 1] doi: 10.21203/rs.3.rs-2587250/v1 (PMC9949243; doi:10.21203/rs.3.rs-2587250/v1)
Supplement: Additional File 1 — Image Segmentation Full Description Detailed description of the process used to segment the vessel and PVS. [file AdditionalFile1.pdf]

Additional File 1: Image Segmentation Full Description

Introduction

To define the boundaries of the vessel and perivascular space, we segmented the images based on tracer intensity. Because the fluorescent signal intensity is attenuated as it passes through red blood cells and tissue, we used a depth-varying intensity threshold, classifying a region as part of the vessel (or PVS) when its intensity exceeded the threshold. For each vessel and PVS, we tried four different approaches to determine the depth-varying threshold: Otsu's method, Edge Finder, Noise Finder, and Mean + Standard Deviation (each described below). After segmenting images according to the threshold, we fine-tuned and compared the resulting segmentation to the original image, as described in the validation section below. For each vessel and PVS, the approach that yielded the closest match between image and segmentation, according to the visual inspection, was selected and validated to quantitatively verify the visual observations.

Approach: Otsu's method

This approach used Otsu's method to determine a threshold that varies with depth using the built-in MATLAB command "graythresh" [1]. Otsu's method maximizes the inter-class variance of intensity between the segmented and non-segmented regions. After applying Otsu's method, we determined the depth where the threshold was maximum and set the threshold equal to this maximum value at all shallower depths.

Approach: Edge Finder

The Edge Finder approach determined the threshold at each depth based on the maximal gradient magnitude. Prior to segmentation, the image was smoothed with a 3D Gaussian filter (using the built-in MATLAB function "imgaussfilt3") with a standard deviation of 2 voxels. At each depth, we identified edges (regions of the maximal gradient magnitude) using Matlab's "edge" command and set the threshold equal to the median intensity value on the edges. The resulting threshold did not always monotonically decrease with depth as we would expect, as shown in Figure S1, Additional File 1 (dashed black line), so we refined the threshold by manually defining, based on the raw signal, a depth-varying threshold that is constant at shallow and deep depths and decreased linearly between the constant regions. Then we compared the resulting segmentation with the original intensity image and iteratively modified the refined threshold until the segmentation qualitatively agreed with the vessel/PVS location in the raw intensity images. The resulting threshold "Edge Finder" is shown in Figure S1, Additional File 1(green line).

Approach: Noise Finder

The noise finder approach determined the threshold based on an assumed distribution of noise in the intensity image at each depth. The intensity distribution of each image had two peaks, one corresponding to noise and one corresponding to the distribution of signal (the upper peak). The optimal threshold for separating signal and noise lies somewhere in between those two peaks. We assumed that the distribution left of the lower peak contained predominantly noise and that the distribution of noise was normal, then fit half a normal curve to the distribution left of the lower peak, determining a mean and standard deviation for the noise. The threshold at each depth was then set

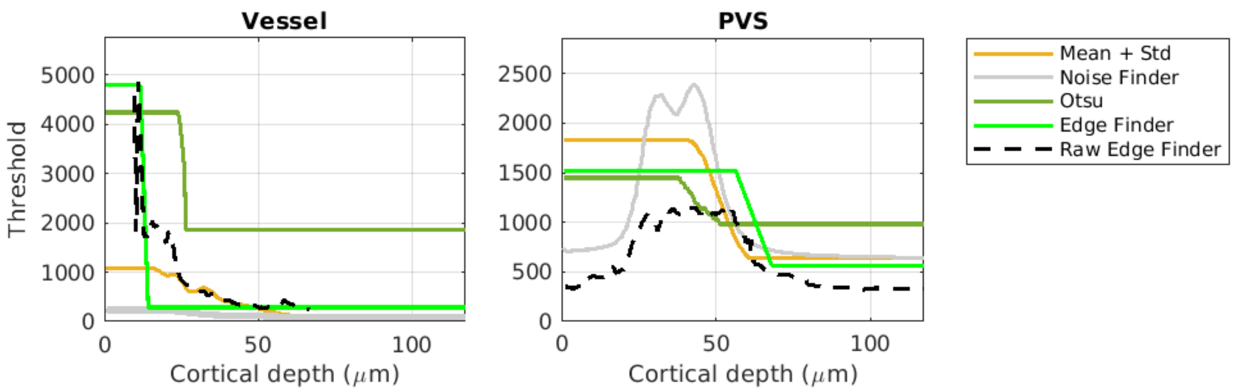

Figure S1, Additional File 1: The depth-varying thresholds used to segment an example vessel and PVS with five methods: Mean + Standard Deviation, Noise Finder, Otsu, Edge Finder, and Raw Edge Finder. In this example case, the Edge Finder method was best because it removed most of the noise and preserved the majority of the region considered to be PVS and vessel, as depicted in Figure S2, Additional File 1.

equal to the mean plus three standard deviations so that 95% of the assumed noise was below the threshold.

Approach: Mean + Standard Deviation

The Mean + Standard Deviation approach set the threshold equal to the sum of the mean and standard deviation of all intensity values at each depth. The depth-varying threshold array was then modified so that the threshold at all depths shallower than the maximum threshold was equal to the maximum.

Performance of segmentation approaches

Approach: Otsu's Method

As shown in Figure S1, Additional File 1, Otsu's method resulted in a high threshold for the vessel because of the presence of other, brighter vessels in the field of view. Thus, the vessel of interest was incorrectly classified as noise, as shown in Figure S2, Additional File 1. Similarly, in images that showed only a single vessel but had a low signal-to-noise ratio, Otsu's method often identified significant portions of the vessel as noise. When the signal-to-noise ratio was high, this approach performed similarly to the Mean + Standard Deviation method but still tended to under-segment the vessel. Since it is easier to remove over-segmented noise with additional post-processing, Otsu's method was not used for segmentation.

Approach: Edge Finder

The Edge Finder approach determines a threshold only in places where the edge is detected, so it is not affected by the absence of a signal. The two-photon imaging signal weakens rapidly through the vessel because the red blood cells attenuate the signal more than the surrounding tissue. The sudden drop in signal helps detect the edges of the vessel, contributing to a more accurate segmentation as indicated in Figure S2, Additional File 1. For the example case shown in Figure S1, Additional File 1, at cortical depth exceeding 15 μm the Edge Finder approach recommended a lower threshold for the vessel than the Mean + Standard Deviation approaches and a higher threshold than the Noise Finder approach. In this case, the Edge Finder approach yielded the most accurate results because it preserved the vessel and removed a large portion of the noise. In general, Edge Finder strikes a good balance between preserving the signal (Mean + Standard Deviation often under-segments, missing critical portion of the vessel or PVS) and removing the noise (Noise Finder over-segments, including

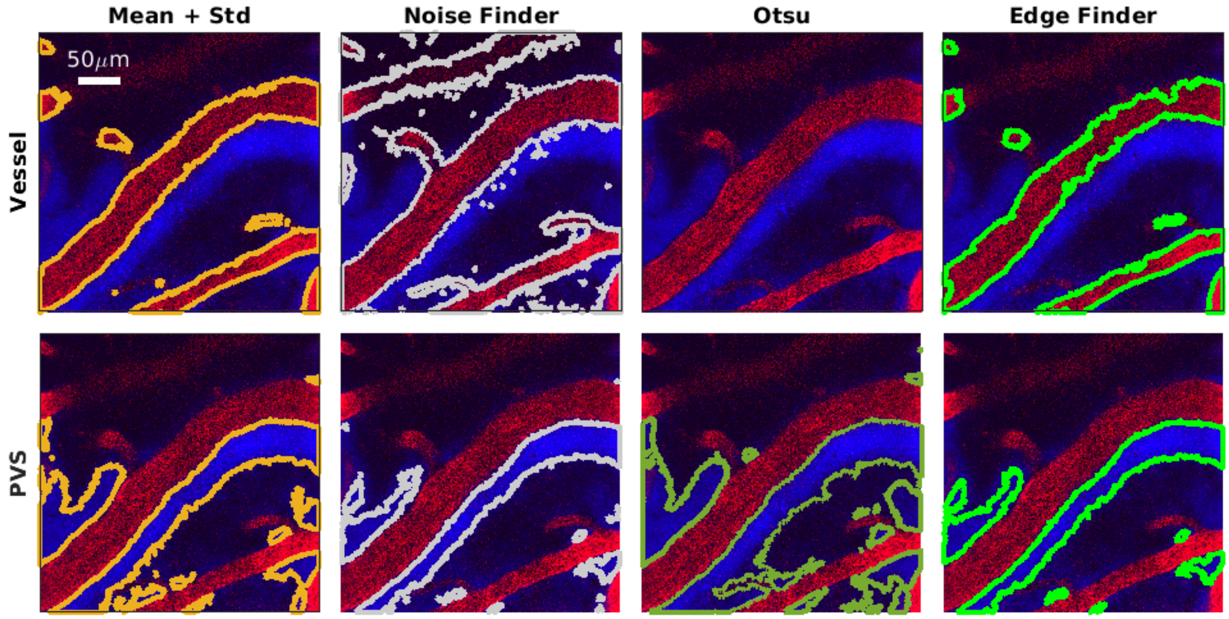

Figure S2, Additional File 1: Segmentations of an example vessel (top row) and PVS (bottom row) at cortical depth 51 μm using four different methods: Mean + Standard Deviation, Noise Finder, Otsu, and Edge Finder. The colored lines indicate the boundary of the segmentation. Of the four, the Edge Finder approach identified the PVS and vessel most accurately.

too much noise). For this reason, the segmentation produced by the Edge Finder was used for this data set. Finally, the method adjusts well to the level of the intensity of the signal independent of the signal-to-noise ratio. The Edge Finder approach also accurately detected the edges of the PVS segmentation in this example case.

Approach: Noise Finder

This method typically is prone to segment noise as shown in Figure S2, Additional File 1. It does well at preserving the signal, but the resulting segmentation tends to overestimate the size of the vessel. The method is best in cases where the signal-to-noise ratio is low since other methods remove a lot of the vessel. The Noise Finder does not perform as robustly as the other approaches when the signal-to-noise ratio is high, segmenting too much noise. This limitation is evident in Figure S2, Additional File 1 and S1, Additional File 1 where the signal is strong.

Approach: Mean + Standard Deviation

The dependence on the mean and standard deviation of the intensity values makes the method prone to inaccuracy when the size of the vessel and PVS are larger relative to the field of view because the approach is based on the assumption that most of the voxels are normally-distributed noise. When the vessel or PVS takes up a larger portion of the field of view, the mean pixel intensity is higher and results in a threshold that is higher than much of the signal and removes too much of the vessel and PVS. In Figure S2, Additional File 1, the Mean + Standard Deviation approach preserves more of the vessel than the Edge Finder approach because of the more rapid decrease in the threshold. The threshold eventually drops below the threshold resulting from the Edge Finder approach, as depicted in Figure S1, Additional File 1. This could be beneficial in cases of lower noise, but looking at cortical depths under 51µm for the data set in Figure S2, Additional File 1, the Edge Finder approach does a much better job at removing the noise and preserving the edges of the vessel.

Selecting the final segmentation

We used all four approaches to segment each data set, then selected the best approach for each case based on a comparison to the original intensity images. In some cases, one approach worked best for the PVS while a different approach worked best for the vessel. The Edge Finder approach resulted in the best segmentation most frequently, but in some cases where the signal-to-noise ratio was low, the edge detection was poor and the Edge Finder approach did not work well. For a few data sets, the Edge Finder approach yielded an accurate representation of PVS but poorly represented the vessel so Mean + Standard Deviation or Noise Finder was used to segment the vessel. The Noise Finder approach often worked well for segmenting the vessel in cases with especially low signal-to-noise ratios. In the case shown in Figure S2, Additional File 1, the Noise Finder approach preserved most of the vessel, but the Edge Finder approach was most effective at removing the noise and leaving only the vessel of interest and accurate representation of PVS. Therefore, for this data set, the Edge Finder approach was used for both PVS and vessel.

References

[1] Nobuyuki Otsu. Threshold selection method from gray-level histograms. *IEEE Trans Syst Man Cybern*, SMC-9, 1979.
