# Supplementary figures and images for: Sizes and Shapes of Perivascular Spaces Surrounding Murine Pial Arteries
Source: Res Sq. 2023 Feb 17:rs.3.rs-2587250. Preprint. [Version 1] doi: 10.21203/rs.3.rs-2587250/v1 (PMC9949243; doi:10.21203/rs.3.rs-2587250/v1)

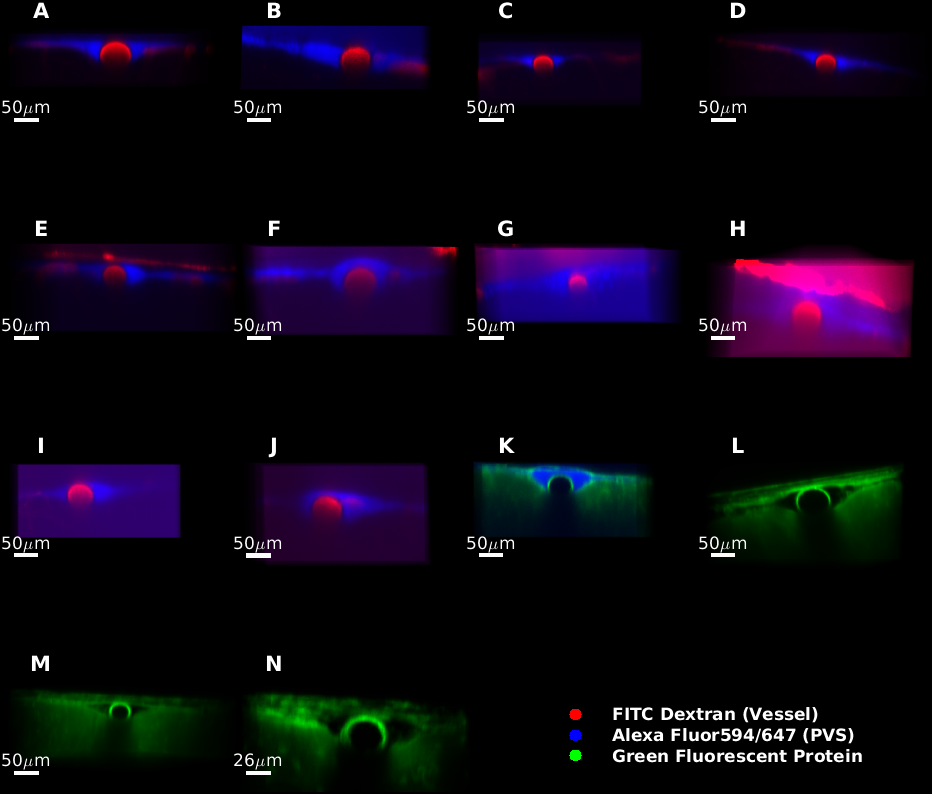

Supplement: Additional File 2 — Intensity Images Averages along the vessel center-line of all of the cross-sectional images at each of the 14 locations. Each panel (A-N) corresponds to a different PVS segment. [file AdditionalFile2.png]

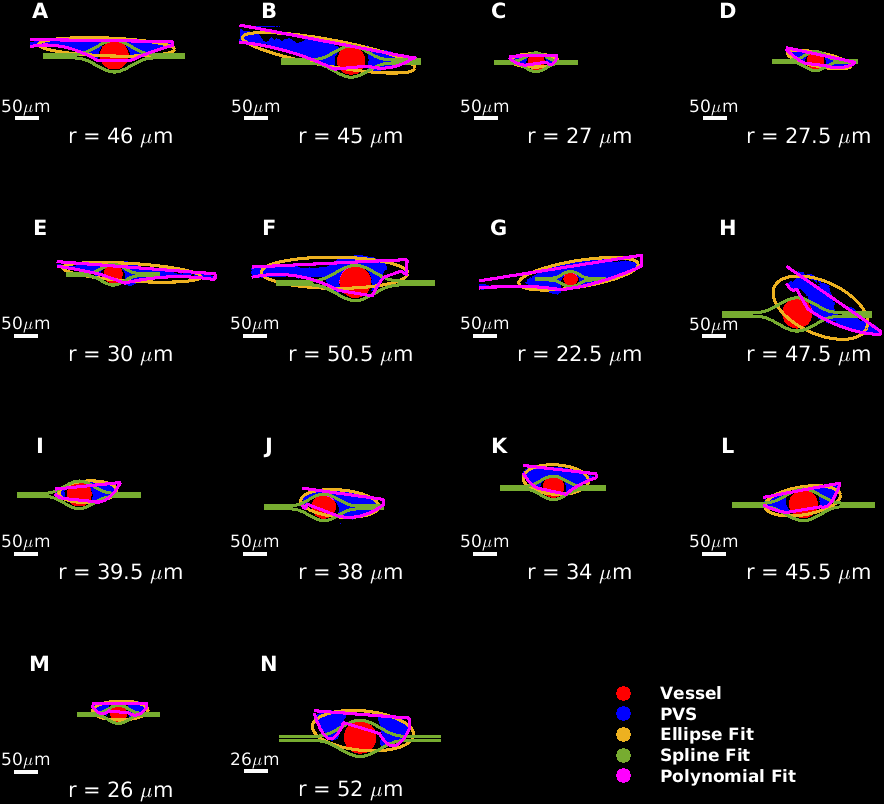

Supplement: Additional File 3 — Segmentation Fits Segmentations and fits of the average images shown in Additional File 2, with vessel radius r indicated. The polynomial fit matched the PVS segmentation most closely in most cases [file AdditionalFile3.png]
